# Exploring the Evolution of Robotic Surgery in Obstetrics and Gynecology: Past, Present, and Future Perspectives

**DOI:** 10.7759/cureus.57155

**Published:** 2024-03-28

**Authors:** Pallavi Yadav, Kamlesh Chaudhari, Apoorva Dave, Arman Sindhu

**Affiliations:** 1 Obstetrics and Gynecology, Jawaharlal Nehru Medical College, Datta Meghe Institute of Higher Education and Research, Wardha, IND; 2 Respiratory Medicine, Jawaharlal Nehru Medical College, Datta Meghe Institute of Higher Education and Research, Wardha, IND

**Keywords:** evolution, technology, minimally invasive, gynecology, obstetrics, robotic surgery

## Abstract

Robotic surgery has emerged as a transformative technology in obstetrics and gynecology, offering enhanced precision and minimally invasive techniques for various procedures. This paper explores the evolution of robotic surgery in obstetrics and gynecology, examining its historical development, current applications, and prospects. Through a comprehensive review of the literature and case studies, we highlight the benefits of robotic surgery, including reduced trauma, improved patient outcomes, and increased surgeon capabilities. However, cost, training requirements, and regulatory concerns hinder widespread adoption. Continuing technological innovation is expected to enhance the efficacy and applicability of robotic-assisted procedures. Collaboration between healthcare professionals, researchers, and industry stakeholders is essential to address these challenges and ensure optimal patient care. By embracing the opportunities presented by robotic surgery while addressing associated challenges, practitioners and researchers can contribute to the continued advancement of this transformative technology in obstetrics and gynecology.

## Introduction and background

Robotic or robot-assisted surgery uses advanced robotic systems to perform surgical procedures. These systems typically consist of robotic arms controlled by surgeons equipped with miniature instruments and a high-definition camera, providing enhanced precision and dexterity during operations. The robotic platform translates the surgeon's hand movements into precise actions within the patient's body, allowing for minimally invasive procedures with improved accuracy [1].

Robotic surgery has emerged as a valuable tool in obstetrics and gynecology, offering numerous benefits to patients and healthcare providers. In gynecological surgery, robots are increasingly used for procedures such as hysterectomies, myomectomies, and ovarian cystectomies, among others. In obstetrics, robotic-assisted techniques are employed for complex surgeries like cesarean sections and fetal interventions [2]. The importance of robotic surgery in this field lies in its ability to minimize trauma, reduce blood loss, shorten hospital stays, and accelerate patient recovery times. Additionally, the enhanced visualization and precision of robotic systems enable surgeons to perform intricate procedures with greater ease and safety [3].

This paper explores robotic surgery's evolution in obstetrics and gynecology, examining its past developments, current applications, and prospects. By delving into the historical background, present state, and potential advancements of robotic surgery in this field, the paper aims to provide insights into the transformative impact of technology on surgical practices. Through a comprehensive analysis of case studies, technological innovations, challenges, and opportunities, this paper seeks to inform healthcare professionals, researchers, and policymakers about the evolving landscape of robotic surgery in obstetrics and gynecology.

## Review

Historical background

Early Developments in Robotic Surgery in Obstetrics and Gynecology

Early advancements in robotic surgery within obstetrics and gynecology can be traced back to the implementation of the da Vinci Surgical System, a pivotal moment that brought about significant changes in surgical practices. This robotic platform offers many advantages, including improved visualization, enhanced dexterity, and the potential for better patient outcomes [4,5]. The inaugural robotic gynecologic procedure was conducted utilizing the Zeus Robotic Surgical System (ZRSS), marking the inception of this sophisticated surgical approach [6]. With the progression of technology, the da Vinci Surgical System emerged as the predominant platform for robotic gynecologic procedures, providing benefits such as minimally invasive surgery and diminished postoperative pain and complications [4]. Integrating robotic surgery into obstetrics and gynecology precipitated a shift in surgical management, with increasingly complex procedures being executed through this advanced technology. This trajectory has continued to expand, with ongoing research and innovations in robotic systems continually augmenting their capabilities and potential for enhancing patient care [4,5,7].

Milestones and Key Advancements

Milestones and critical advancements in robotic gynecologic surgery have been instrumental in shaping the progress of the field. Historical breakthroughs in surgical technology, originating from gynecology, have laid the foundation for the transition to robotic-assisted surgery. The evolution has been remarkable from early innovations such as Hippocrates' utilization of tin catheters to the emergence of modern robotic systems [8]. One notable milestone is the infusion of inspiration from the National Aeronautics and Space Administration (NASA) into some of the most significant advances in robotic surgery technology, highlighting the interdisciplinary nature of innovation in gynecologic surgery [6]. The application of robotics in surgery has played a pivotal role in propelling medical technology forward, with robotics now firmly entrenched as an integral component of surgical specialties [9].

A comprehensive review of the literature underscores the pivotal role of robotic surgery in driving notable advancements in medical technology in recent decades. This review underscores the concept of robotic surgery as a transformative force, enhancing surgical precision and improving patient outcomes [9]. Together, these milestones and advancements underscore the transformative impact of robotic surgery on gynecologic procedures, offering enhanced precision, improved dexterity, and the potential for better patient outcomes. Integrating robotics into gynecologic surgery represents a significant leap forward in surgical techniques and patient care [6,8,9].

Impact of Robotic Surgery on the Field

Robotic surgery has profoundly impacted the field of gynecology, ushering in advancements in surgical techniques and elevating patient outcomes. Studies indicate that robot-assisted gynecologic surgery, when conducted by experienced surgeons, can be performed safely, with perioperative outcomes akin to those of traditional methods [10]. Moreover, robotic surgery has exhibited superiority over open surgeries in reducing post-surgical hospital stays, underscoring its potential for enhancing recovery times and patient experiences [6].

The utilization of robotic systems in gynecological surgery has undergone continual evolution, with ongoing research endeavors aimed at elucidating the trends and patterns of robotic surgery in obstetrics and gynecology. This research endeavor strives to discern the advantages and constraints of robotic surgery relative to conventional techniques, offering insights into the prospective trajectory of this field [7]. Collectively, robotic surgery has left a significant imprint by augmenting surgical precision, enhancing visualization, and potentially fostering improved patient care outcomes in gynecologic procedures [6,7,10,11].

Present state of robotic surgery in obstetrics and gynecology

Current Applications and Procedures

Hysterectomy: Robotic-assisted hysterectomy stands as a frequently employed procedure for the removal of the uterus, typically undertaken to address benign conditions like uterine fibroids or endometriosis [10]. This minimally invasive approach offers several advantages over traditional methods, including smaller incisions, reduced blood loss, and faster recovery times. By harnessing robotic technology, surgeons can navigate intricate anatomical structures with heightened precision, thereby minimizing the risk of complications and optimizing patient outcomes.

Myomectomy: Robotic-assisted myomectomy excises fibroids from the uterus while preserving the organ for future fertility prospects [10]. This approach provides a delicate balance between thorough fibroid removal and maintaining reproductive capabilities, making it an appealing option for patients seeking both symptom relief and the possibility of conceiving. The robotic platform facilitates meticulous tissue dissection and suturing, enabling surgeons to execute precise maneuvers within the confined pelvic space, ultimately enhancing surgical success rates and patient satisfaction.

Tubal reanastomosis: Robotic-assisted tubal reanastomosis is a technique employed to reconnect the fallopian tubes following a tubal ligation procedure, restoring the potential for future fertility [12]. By leveraging robotic instrumentation, surgeons can meticulously manipulate delicate tubal structures with enhanced precision and control, facilitating accurate anastomosis and optimizing reproductive outcomes. This minimally invasive approach offers patients a less invasive alternative to traditional open surgery, resulting in reduced postoperative pain and shorter recovery periods.

Sacrocolpopexy: Robotic-assisted sacrocolpopexy serves as a surgical intervention for correcting pelvic organ prolapse by suspending the uterus and vagina to the sacrum [13]. This procedure addresses the anatomical defects underlying pelvic organ prolapse while minimizing the risk of recurrence. The robotic platform enables surgeons to navigate complex pelvic anatomy with improved visualization and dexterity, facilitating precise placement of mesh implants and ensuring optimal pelvic support. Consequently, patients experience enhanced symptom relief and restored pelvic function following surgery.

**Figure 1:**
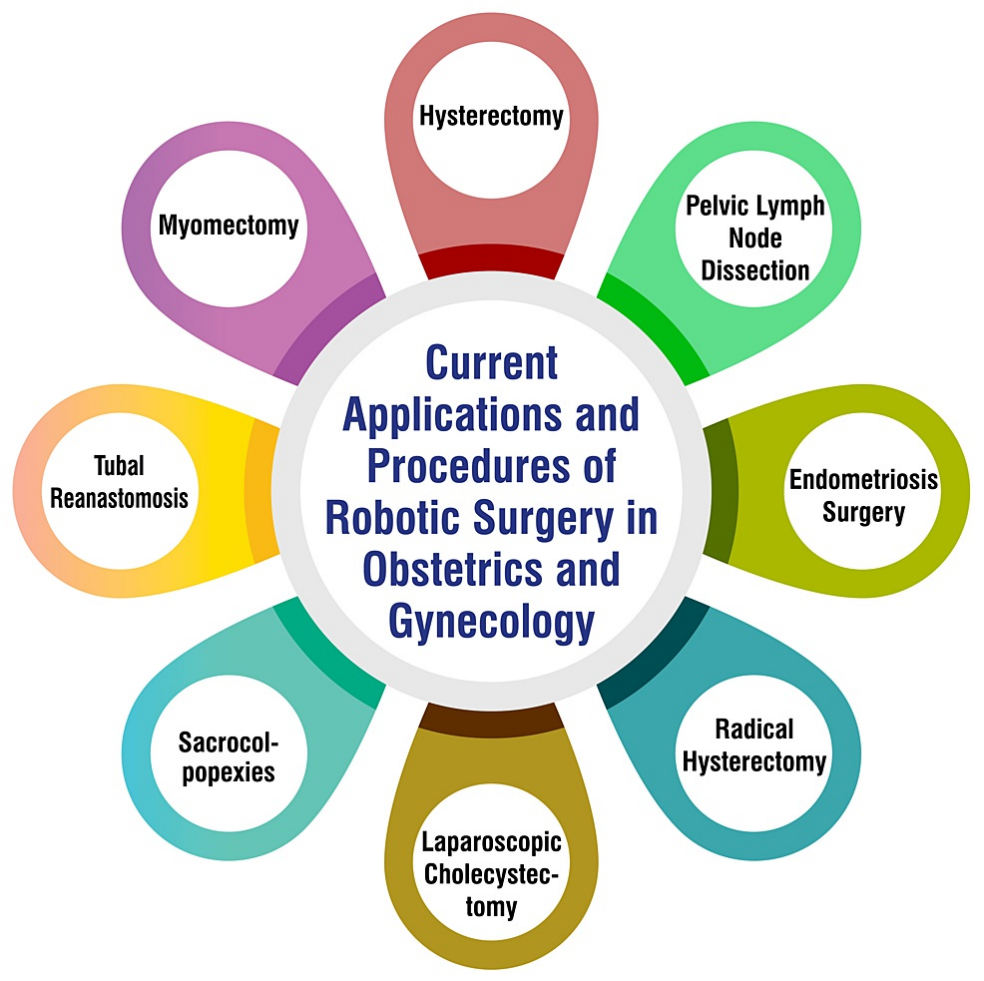
Current applications and procedures

Laparoscopic sleeve gastrectomy: While not directly associated with obstetrics and gynecology, robotic-assisted laparoscopic sleeve gastrectomy has gained popularity as a complementary procedure in conjunction with gynecologic surgeries [14]. This weight loss procedure involves the removal of a portion of the stomach to reduce its capacity, thereby promoting weight loss and improving metabolic health. By integrating robotic technology into the surgical approach, surgeons can achieve greater precision and efficiency during sleeve gastrectomy, improving outcomes and patient satisfaction. Current applications and procedures are shown in Figure 1.

Advantages

Robotic surgery offers many benefits that have transformed the landscape of surgical practice in gynecology. Firstly, it enhances the operative field's visualization, facilitating more precise and accurate procedures [10]. Surgeons benefit from a more transparent and magnified view of anatomical structures, enabling them to confidently navigate intricate surgical pathways. This heightened visual clarity contributes to improved surgical outcomes and patient safety. Secondly, robotic surgery enhances surgeon dexterity through the robot's arms, which provide greater precision and control [10]. Surgeons can perform complex procedures with increased ease and efficiency, achieving optimal results while minimizing the risk of errors. The intuitive motion-scaling technology enables precise tissue manipulation, enabling surgeons to navigate delicate anatomical structures precisely.

Moreover, robotic surgery eliminates the effects of surgeon tremors by providing steady robotic arms, thereby reducing the risk of complications [10]. Surgeons can execute precise maneuvers without the interference of involuntary movements, ensuring consistent instrument control and minimizing tissue trauma. This feature enhances surgical accuracy and patient safety, particularly in delicate procedures.

Furthermore, the ergonomic design of the robotic platform reduces surgeon fatigue and strain, leading to a more comfortable surgical experience [10]. Surgeons operate seated at a console, with ergonomic hand controls and adjustable display settings, minimizing physical discomfort during prolonged procedures. This ergonomic optimization promotes surgeon well-being and enhances overall surgical performance.

**Figure 2:**
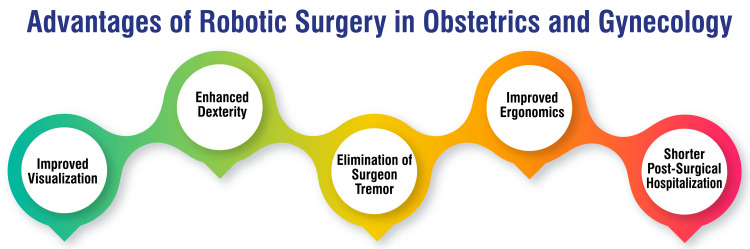
Advantages of robotic surgery in obstetrics and gynecology

Beyond these technical advantages, robotic-assisted gynecologic procedures offer significant clinical benefits. They are associated with reduced postoperative pain compared to traditional laparoscopic or open surgical techniques [15]. Patients undergoing robotic surgery often experience shorter hospital stays due to faster recovery times, allowing for a quicker return to normal activities and daily routines [15]. Some studies suggest that adopting robotic surgery can lead to cost reductions for healthcare institutions as the incidence of laparotomy declines [16]. Additionally, robotic surgery enables less experienced laparoscopic surgeons to perform more complex procedures, improving patient care and expanding access to advanced surgical techniques [4]. Overall, robotic surgery has revolutionized the field of gynecology, offering unparalleled precision, improved patient outcomes, and enhanced surgical capabilities. The advantages of robotic surgery in obstetrics and gynecology are shown in Figure 2.

Limitations

Robotic surgery in obstetrics and gynecology, while offering numerous advantages, also presents challenges and limitations that impact its widespread adoption and utilization. Firstly, the high cost of robotic surgery represents a significant barrier, as it is notably higher than traditional surgical techniques, potentially limiting access for patients and healthcare institutions [4,17]. Additionally, a steep learning curve is associated with robotic surgery, requiring surgeons to undergo extensive training to attain proficiency. This learning curve can result in longer operative times and increased costs during the initial stages of implementation [4,17]. Moreover, the need for more high-quality evidence and long-term data comparing the outcomes of robotic surgery with traditional techniques poses a challenge [4,17]. While robotic surgery offers improved visualization and dexterity, it may not necessarily translate into shorter operative times compared to open or conventional laparoscopic techniques [4,17]. Furthermore, the maintenance and calibration of robotic equipment add to surgical procedures' complexity and overall cost [18].

Accessibility to robotic surgery may be limited due to its high cost and restricted availability in certain regions or healthcare systems, contributing to disparities in patient access to advanced surgical care [19]. Like any surgical procedure, robotic surgery risks complications such as infection, bleeding, or damage to surrounding tissues [20]. The need for standardization in robotic surgery techniques further complicates matters, leading to surgical approach and outcome inconsistencies [21]. While robotic surgery offers considerable advantages in obstetrics and gynecology, including improved precision and patient outcomes, it also presents significant challenges that must be addressed to optimize its utilization and accessibility in clinical practice. Efforts to mitigate these challenges, such as reducing costs, enhancing training programs, and standardizing surgical techniques, are essential to realize the full potential of robotic surgery in improving patient care.

Comparative Analysis With Traditional Methods

A comparative analysis between robotic surgery and traditional methods in obstetrics and gynecology reveals distinct advantages inherent in each approach. Robotic surgery, characterized by greater precision, enhanced visibility, and shorter recovery times, presents a compelling alternative to traditional methods [22]. Its minimally invasive nature translates to reduced risk factors and complications commonly associated with larger incisions, thereby improving patient outcomes [23]. Conversely, traditional surgery offers a more hands-on approach but may entail heightened pain levels and more extended recovery periods [24]. Regarding surgical outcomes, robotic surgery often yields fewer complications and a lower risk of infection, rendering it a favorable choice for many patients undergoing gynecological procedures [25]. The future landscape of gynecology is increasingly incorporating robotic surgery due to its significant superiority in various benign and malignant gynecological surgeries, underscoring its transformative potential in advancing patient care [6].

Technological innovations and future trends

Emerging Technologies in Robotic Surgery

The development of surgical robots is a revolutionary advancement in the medical field, facilitating precise and minimally invasive procedures across various medical specialties [26]. These sophisticated robotic systems have transformed surgical practices, offering surgeons unprecedented control and accuracy during complex operations. By leveraging robotic technology, surgeons can perform intricate procedures with enhanced precision, leading to improved patient outcomes and reduced recovery times. Robotic systems are increasingly utilized to precisely navigate catheters and endoscopes, enhancing procedural accuracy and outcomes [27]. This advanced technology enables surgeons to maneuver catheters and endoscopes with unparalleled precision, ensuring optimal placement and trajectory during diagnostic and interventional procedures. Integrating robotic navigation systems enhances procedural success rates and minimizes the risk of complications, ultimately improving patient care and safety.

Automation in positioning surgical tools using robotic systems has emerged as a significant advancement, enhancing efficiency and precision during surgeries [27]. Robotic-assisted positioning of surgical instruments enables surgeons to execute precise maneuvers with greater ease and accuracy, minimizing the risk of errors and optimizing surgical outcomes. This automation streamlines surgical procedures, allowing surgeons to focus on critical aspects of the operation while ensuring consistent instrument placement and movement. Robotic systems for intraoperative camera manipulation offer surgeons enhanced visualization and control during procedures [27]. These sophisticated robotic platforms enable real-time adjustment and manipulation of the surgical camera, providing surgeons with a clear and detailed view of the operative field. By optimizing visualization, these systems facilitate accurate tissue identification, precise instrument placement, and meticulous surgical technique, ultimately improving procedural efficiency and patient outcomes.

**Figure 3:**
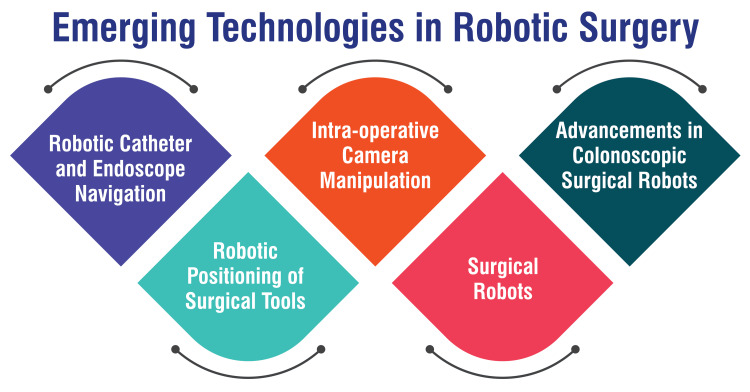
Emerging technologies in robotic surgery

Recent innovations in colonoscopic surgical robots have focused on improving key areas such as adjustable stiffness, detectability, and bendability, enhancing the effectiveness of these procedures [28]. These advancements aim to overcome challenges associated with traditional colonoscopic techniques, offering improved maneuverability and visualization within the colon. By enhancing the flexibility and functionality of colonoscopic surgical robots, these innovations promise to enhance these procedures' diagnostic and therapeutic capabilities, leading to improved patient outcomes and quality of care. Emerging technologies in robotic surgery are shown in Figure 3.

Potential Advancements on the Horizon

Miniaturization and micro-robotics: Engineers are at the forefront of developing miniaturized robots, commonly called microbots, characterized by their smaller size, increased speed, and enhanced efficiency compared to traditional robotic systems [29]. These microbots represent a significant advancement in surgical technology, offering a range of applications to reduce surgical procedures' invasiveness further. With their diminutive size, microbots can access anatomical structures and navigate intricate pathways within the body with unprecedented precision. This capability allows for minimally invasive surgeries where traditional instruments may be too large or cumbersome. By leveraging micro-robotics, surgeons can perform procedures with greater precision and accuracy, minimizing tissue trauma and accelerating patient recovery times.

Artificial intelligence (AI) and machine learning: The integration of AI and machine learning algorithms into robotic surgery is poised to play an increasingly prominent role in the field [30]. These cutting-edge technologies have the potential to revolutionize surgical practices by assisting in real-time decision-making, optimizing surgical planning, and enhancing the precision of robotic procedures. AI algorithms can analyze vast amounts of patient data and surgical outcomes to identify patterns and trends, enabling surgeons to make more informed decisions during procedures. Machine learning algorithms can also adapt and improve over time based on experience, further enhancing their utility in robotic surgery. Robotic systems can continuously evolve to deliver superior surgical outcomes and enhance patient safety by harnessing AI and machine learning.

Telepresence in surgery and global translation: The advancement of remote surgery, also known as telesurgery or telepresence, is expected to continue expanding, allowing surgeons to perform procedures on patients in different geographic locations [31]. This transformative trend has the potential to revolutionize healthcare delivery by enhancing access to high-quality surgical care, particularly in underserved or remote areas. Telepresence technology enables surgeons to remotely control robotic systems equipped with surgical instruments, cameras, and other tools, allowing them to perform procedures with precision and expertise from afar. Additionally, global translation technology facilitates communication between surgeons and patients, overcoming language barriers and ensuring effective collaboration during remote surgeries. By leveraging telepresence technology, healthcare providers can extend their reach and expertise to patients worldwide, ultimately improving overall health outcomes and reducing healthcare disparities.

Ethical and Societal Considerations

The advancement of robotic surgery in obstetrics and gynecology brings many ethical and societal considerations. The increased utilization of robotic-assisted surgeries has prompted ethical inquiries regarding the quality of care provided, informed decision-making processes, potential conflicts of interest, innovation in healthcare practices, and the ongoing education of medical professionals. The American Medical Association (AMA) Code of Medical Ethics offers pertinent guidelines concerning robotic surgery, emphasizing the imperative of ensuring safe, effective, patient-centered, timely, efficient, and equitable care [32]. Moreover, integrating AI into medicine and healthcare introduces its own ethical challenges. Concerns arise regarding the potential diminishment of compassion and empathy in clinical settings when interacting with robotic doctors and nurses. Patients may encounter difficulties accepting machine-human medical relations over traditional human-human interactions, adversely impacting healing [33].

From a medicolegal standpoint, robotic surgery presents challenges related to standardized training protocols, informed consent procedures, and legal liabilities due to the involvement of multiple stakeholders. Developing comprehensive guidelines, regulations, and training programs is essential to effectively navigate the medicolegal aspects associated with robotic surgery and unlock its full potential for the future [34]. Despite the numerous advantages of robotic surgery, such as enhanced precision and reduced surgeon fatigue compared to traditional methods, there are also drawbacks, such as the loss of haptic feedback (touch). As robotic technology continues to evolve, addressing ethical dilemmas, ensuring patient safety through appropriate training and guidelines, and delineating legal responsibilities for malfunctions are pivotal steps for the future of robotic surgery in gynecology [35].

Challenges and barriers to adoption

Cost Implications

The cost implications of robotic surgery in obstetrics and gynecology play a pivotal role in shaping its adoption and utilization within healthcare systems. Evaluating the cost-effectiveness of robotic surgery is crucial, focusing on implementing strategies and considering factors that maximize efficiency and minimize expenses to facilitate its widespread implementation [36]. Studies underscore the challenges in assessing costs associated with robotic surgery, underscoring the necessity for enhanced costing methodologies to provide accurate insights into the economic implications of these procedures [37].

Recent research indicates that the total operating room costs for common general surgery procedures before introducing robotic surgery ranged from $3000 to $7000. However, in 2017, hospitals allocated over $3 billion to the primary supplier for robotic technology, equating to an average cost of approximately $3568 per procedure. This breakdown of costs includes $1866 for instruments and accessories, $1038 for robot systems, and $663 for service contracts, reflecting the substantial financial investment required for hospitals to adopt and maintain robotic surgical systems [38].

Additionally, additional expenses such as staff training, infrastructure upgrades, and marketing efforts contribute to the overall cost of implementing robotic surgery in obstetrics and gynecology practices. While potential downstream benefits such as reduced length of stay may offset some of these costs, it is essential to recognize that the financial implications of robotic surgery extend beyond equipment and maintenance expenses alone. Therefore, a comprehensive understanding of the detailed cost structure associated with robotic surgery is imperative for healthcare facilities to make well-informed decisions regarding its integration into obstetrics and gynecology practices [38].

Training and Education Requirements

Training and education requirements for robotic surgery in obstetrics and gynecology entail a structured curriculum to cultivate proficiency and ensure safety in executing fundamental robotic skills. This training regimen typically progresses through several stages, commencing with observation, case assisting, and acquiring basic robotic skills in dry and wet lab settings. Subsequently, trainees advance to individual and team-based non-modular console training under supervision, culminating in independent practice [39,40]. A comprehensive training program encompasses patient-side training, including tasks such as patient positioning, pneumoperitoneum, procedure-specific port placement, robot docking, and basic laparoscopic skills. Concurrently, console training involves simulation exercises in dry and wet labs, supervised practice sessions, and the development of non-technical skills essential for novice surgeons. The curriculum establishes a validated proficiency benchmark before trainees can progress to hands-on experience in the operating room [39,40]. Efforts are underway to standardize robotic surgery training across various specialties, including general surgery. These initiatives underscore the importance of incorporating didactic elements, simulation training, hands-on practice, and expert mentorship systematically and sequentially. Evaluating residents' robotic skills within the operating room environment using structured curricula facilitates the monitoring of their progress and certifies their ability to perform robot-assisted surgery safely [39].

Regulatory Challenges and Safety Concerns

Robotic surgery in obstetrics and gynecology presents various challenges, ranging from technical issues to ethical and legal considerations. Technical failures, including hardware malfunctions and software glitches, pose risks of interruptions or complications during surgery, necessitating stringent maintenance protocols and redundancy measures in healthcare facilities to mitigate these risks [26]. Surgeons must also anticipate and manage intraoperative challenges, such as robotic arm malfunctions or communication issues between the surgeon and the console [41]. Moreover, introducing robotic systems introduces new infection risks if not properly sterilized between procedures. Establishing and strictly adhering to proper cleaning and sterilization protocols following manufacturer guidelines and best practices is imperative to minimize infection risks [42]. Despite advancements, robotic surgical systems still lack complete haptic feedback, requiring surgeons to rely on visual and auditory cues and their expertise to compensate [26].

Ethical and legal challenges arise with integrating AI and autonomous features in robotic surgery, particularly regarding liability in case of errors or malfunctions. Clear guidelines and frameworks defining responsibility and liability in such cases need to be established by healthcare institutions and regulatory bodies [43]. Additionally, privacy concerns regarding the collection and transmission of patient data during telesurgery highlight the importance of establishing ethical standards and legal regulations to safeguard patient data throughout the process [44]. Patient acceptance of robotic surgery may be hindered by limited technology awareness, emphasizing the need for healthcare providers to prioritize patient education and awareness campaigns [45]. Addressing patients' concerns about the safety and effectiveness of robotic surgery is essential for promoting acceptance, requiring open and transparent communication from healthcare providers [26]. Disparities in access to information about robotic surgery can impact decision-making, highlighting the importance of providing accessible and patient-friendly resources that explain its benefits [46].

**Figure 4:**
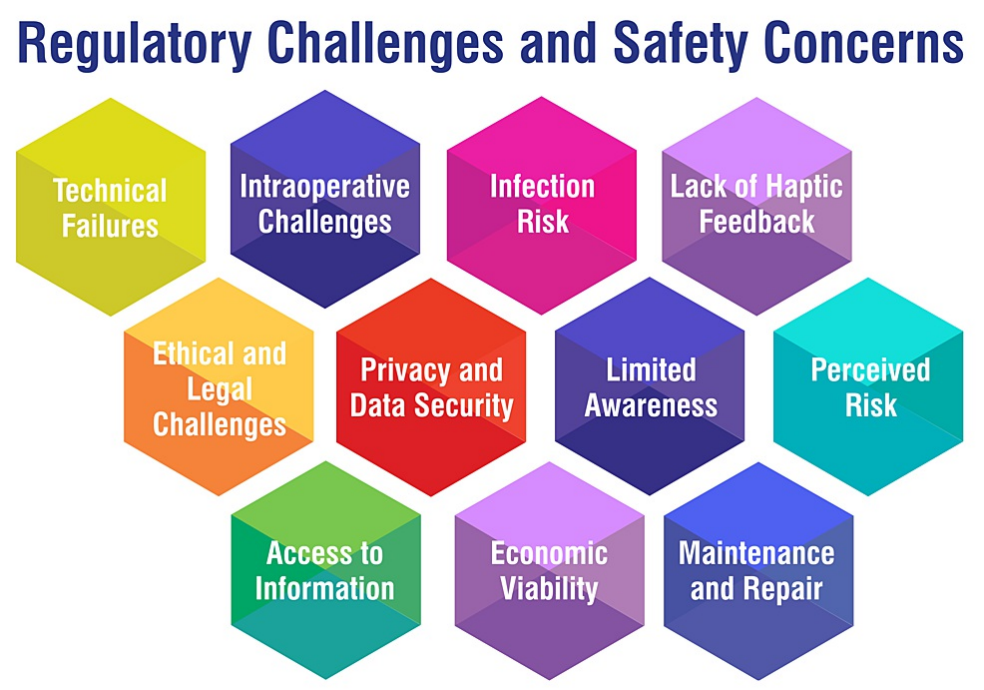
Regulatory challenges and safety concerns

Economic considerations, including the high cost of robotic surgery equipment, pose barriers to widespread adoption, with upfront expenses being a concern for many healthcare facilities [47]. Surgeons must undergo extensive training to become proficient in robotic surgery techniques, which can impact patient safety during the learning curve [48]. Regular equipment inspections, software updates, and backup systems are crucial for minimizing the impact of technical failures on patient safety [49]. Addressing these challenges is essential for maximizing the benefits of robotic surgery while ensuring patient safety and ethical practice in obstetrics and gynecology. Regulatory challenges and safety concerns are shown in Figure 4.

Opportunities for research and collaboration

Areas for Further Study and Investigation

Numerous collaborative research programs and institutions spearhead robotic surgery advancements and medical technology. Initiatives such as the National Science Foundation's (NSF's) AccelNet program serve as platforms for coordination, dissemination, workshops, tutorials, and surgical robotics challenges, facilitating the advancement of research in the field [50]. Institutions like Children's Hospital actively participate in over 40 collaborative research projects to advance minimally invasive technology for pediatric surgery [51]. Additionally, laboratories specializing in computational sensing and robotics provide opportunities for faculty and students to collaborate in developing and testing innovative robotic systems for surgical applications [51]. The Biomechanical- and Image-Guided Surgical Systems (BIGSS) Laboratory focuses on developing computer-aided surgical guidance systems utilizing novel robots, advanced imaging techniques, and real-time biomechanical assessments to enhance surgical outcomes [51]. Similarly, the Photoacoustic and Ultrasonic Systems Engineering (PULSE) Lab integrates light, sound, and robotic technologies to develop innovative biomedical imaging systems addressing clinical needs in neurosurgery, cancer detection, and women's health [51]. The Medical UltraSound Imaging and Intervention Collaboration (MUSiiC) aims to develop ultrasound technologies for medical applications such as prostate and breast cancer treatment through collaboration among researchers from various institutions [51].

Exploring the human perception of human-robot interaction in medical robotics applications, the Haptics and Medical Robotics (HAMR) Lab investigates areas like minimally invasive surgical robots, prosthetic devices, and rehabilitation robots [51]. The Locomotion in Mechanical and Biological Systems (LIMBS) Laboratory delves into animal and robot sensory guidance principles to enhance robot control strategies for sensing, navigation, and control applications [51]. Meanwhile, the Computational Interaction and Robotics Lab (CIRL) focuses on dynamic spatial interaction, intersecting imaging, robotics, and human-computer interaction, with projects like skill evaluation in surgery using robotic systems [51]. Innovative interactive robot systems are developed in the Intuitive Computing Laboratory, integrating human-computer interaction, robotics, and machine learning principles to provide personalized support in healthcare, education, and collaborative manufacturing [51]. Finally, the Advanced Medical Instrumentation and Robotics (AMIRo) Research Laboratory conducts research to support robotic-assisted medical technology for diagnosis, therapy, and clinical research applications [51]. These collaborative efforts and research institutions play a vital role in advancing the field of robotic surgery and medical technology, paving the way for innovative solutions in healthcare.

## Conclusions

The exploration of the evolution of robotic surgery in obstetrics and gynecology reveals a dynamic landscape characterized by significant advancements, challenges, and opportunities. With its precision and minimally invasive nature, robotic surgery has revolutionized surgical practices in the field, offering benefits such as reduced trauma, improved patient outcomes, and enhanced surgeon capabilities. Looking to the future, continued technological innovation is expected further to enhance the efficacy and applicability of robotic-assisted procedures, expanding the scope of treatment for complex obstetric and gynecological conditions. However, challenges such as cost implications, training requirements, and regulatory concerns remain pertinent and require collaborative efforts from practitioners, researchers, and industry stakeholders. Healthcare professionals must stay abreast of the latest developments in robotic surgery and actively engage in training programs to ensure proficiency in robotic-assisted techniques. Additionally, ongoing evaluation of patient outcomes and safety profiles is crucial to maintain the highest standards of care. By embracing the opportunities presented by robotic surgery while addressing the associated challenges, practitioners and researchers can contribute to the continued advancement and integration of this transformative technology in obstetrics and gynecology, ultimately improving patient care and outcomes.

## References

[1] Lanfranco AR, Castellanos AE, Desai JP, Meyers WC (2004). Robotic surgery: a current perspective. Ann Surg.

[2] Gala RB, Margulies R, Steinberg A (2014). Systematic review of robotic surgery in gynecology: robotic techniques compared with laparoscopy and laparotomy. J Minim Invasive Gynecol.

[3] Sinha R, Sanjay M, Rupa B, Kumari S (2015). Robotic surgery in gynecology. J Minim Access Surg.

[4] Weinberg L, Rao S, Escobar PF (2011). Robotic surgery in gynecology: an updated systematic review. Obstet Gynecol Int.

[5] Chen CC, Falcone T (2009). Robotic gynecologic surgery: past, present, and future. Clin Obstet Gynecol.

[6] Chandrakar I, Pajai S, Toshniwal S (2022). Robotic surgery: the future of gynaecology. Cureus.

[7] Levin G, Siedhoff M, Wright KN (2023). Robotic surgery in obstetrics and gynecology: a bibliometric study. J Robot Surg.

[8] (2024). History of minimally invasive surgery in gynecology: Transition to robotic surgery. Basicmedical Key.

[9] Rivero-Moreno Y, Echevarria S, Vidal-Valderrama C (2023). Robotic surgery: a comprehensive review of the literature and current trends. Cureus.

[10] (2020). Robot-assisted surgery for noncancerous gynecologic conditions: ACOG committee opinion, number 810. Obstet Gynecol.

[11] Nobbenhuis MA, Gul N, Barton-Smith P, O'Sullivan O, Moss E, Ind TE (2023). Robotic surgery in gynaecology: Scientific Impact Paper No. 71 (July 2022). BJOG.

[12] (2024). Fallopian tube (tubal) reversal and its impact on fertility. https://www.novaivffertility.com/blog/fallopian-tube-tubal-reversal-and-its-impact-fertility.

[13] (2024). Robotic-assisted sacrocolpopexy. https://www.hopkinsmedicine.org/health/treatment-tests-and-therapies/roboticassisted-sacrocolpopexy.

[14] (2024). Laparoscopic sleeve gastrectomy overview. https://www.hopkinsmedicine.org/health/treatment-tests-and-therapies/laparoscopic-sleeve-gastrectomy-overview.

[15] (2024). 5 benefits of performing gynecologic surgery with robotic assistance. https://www.panhandleobgyn.com/post/5-benefits-of-performing-gynecologic-surgery-with-robotic-assistance.

[16] Truong M, Kim JH, Scheib S, Patzkowsky K (2016). Advantages of robotics in benign gynecologic surgery. Curr Opin Obstet Gynecol.

[17] Bankar GR, Keoliya A (2022). Robot-assisted surgery in gynecology. Cureus.

[18] Berkelman P, Ma J (2009). A compact modular teleoperated robotic system for laparoscopic surgery. Int J Rob Res.

[19] Mehta A, Cheng Ng J, Andrew Awuah W (2022). Embracing robotic surgery in low- and middle-income countries: potential benefits, challenges, and scope in the future. Ann Med Surg (Lond).

[20] Patel N, Chaudhari K, Jyotsna G, Joshi JS (2023). Surgical frontiers: a comparative review of robotics versus laparoscopy in gynecological interventions. Cureus.

[21] Chen IA, Ghazi A, Sridhar A, Stoyanov D, Slack M, Kelly JD, Collins JW (2021). Evolving robotic surgery training and improving patient safety, with the integration of novel technologies. World J Urol.

[22] Köckerling F (2014). Robotic vs. standard laparoscopic technique - what is better?. Front Surg.

[23] (2024). Robotic surgery vs. traditional surgery. https://www.drklause.com/blog/robotic-surgery-vs-traditional-surgery.

[24] Scott MJ, Baldini G, Fearon KC (2015). Enhanced recovery after surgery (ERAS) for gastrointestinal surgery, part 1: pathophysiological considerations. Acta Anaesthesiol Scand.

[25] (2024). Robotic surgery vs traditional surgery: Which is right for you?. https://www.reidhealth.org/blog/robotic-surgery-vs-traditional-surgery-which-is-right-for-you.

[26] Reddy K, Gharde P, Tayade H, Patil M, Reddy LS, Surya D (2023). Advancements in robotic surgery: a comprehensive overview of current utilizations and upcoming frontiers. Cureus.

[27] (2024). Innovations in robotic surgery 2020-2030: Technologies, players & markets. https://www.idtechex.com/en/research-report/innovations-in-robotic-surgery-2020-2030-technologies-players-and-markets/724.

[28] MD DLT (2024). Recent advances in robotic surgery. https://www.news-medical.net/health/Recent-Advances-in-Robotic-Surgery.aspx.

[29] (2024). The future of robotic surgery: 3 trends to look for. https://blog.engineering.vanderbilt.edu/the-future-of-robotic-surgery-3-trends-to-look-for.

[30] Pakkasjärvi N, Luthra T, Anand S (2023). Artificial intelligence in surgical learning. Surgeries.

[31] Avgousti S, Christoforou EG, Panayides AS (2016). Medical telerobotic systems: current status and future trends. Biomed Eng Online.

[32] Young J (2023). AMA Code of Medical Ethics’ opinions related to robotic surgery. AMA J Ethics.

[33] Farhud DD, Zokaei S (2021). Ethical issues of artificial intelligence in medicine and healthcare. Iran J Public Health.

[34] Pai SN, Jeyaraman M, Jeyaraman N, Nallakumarasamy A, Yadav S (2023). In the hands of a robot, from the operating room to the courtroom: the medicolegal considerations of robotic surgery. Cureus.

[35] Lenihan JP (2023). Robotic surgery in gynecology: indications, advantages, avoiding complications, training, and future platforms; update 2022. Handbook of Gynecology.

[36] De Nagy J, Youssef Y, Moawad G (2023). Strategies and factors to maximize cost-effectiveness of robotic surgery in benign gynecological disease. Best Pract Res Clin Obstet Gynaecol.

[37] Korsholm M, Sørensen J, Mogensen O, Wu C, Karlsen K, Jensen PT (2018). A systematic review about costing methodology in robotic surgery: evidence for low quality in most of the studies. Health Econ Rev.

[38] Childers CP, Maggard-Gibbons M (2018). Estimation of the acquisition and operating costs for robotic surgery. JAMA.

[39] Moit H, Dwyer A, De Sutter M, Heinzel S, Crawford D (2019). A standardized robotic training curriculum in a general surgery program. JSLS.

[40] Sridhar AN, Briggs TP, Kelly JD, Nathan S (2017). Training in robotic surgery-an overview. Curr Urol Rep.

[41] Cristancho SM, Vanstone M, Lingard L, LeBel ME, Ott M (2013). When surgeons face intraoperative challenges: a naturalistic model of surgical decision making. Am J Surg.

[42] (2024). Guideline for disinfection and sterilization in healthcare facilities (2008). https://www.cdc.gov/infectioncontrol/guidelines/disinfection/introduction.html.

[43] Gerke S, Minssen T, Cohen G (2020). Ethical and legal challenges of artificial intelligence-driven healthcare. Artificial Intelligence in Healthcare.

[44] Nittari G, Khuman R, Baldoni S (2020). Telemedicine practice: review of the current ethical and legal challenges. Telemed J E Health.

[45] Lawrie L, Gillies K, Duncan E, Davies L, Beard D, Campbell MK (2022). Barriers and enablers to the effective implementation of robotic assisted surgery. PLoS One.

[46] Randell R, Alvarado N, Honey S (2015). Impact of robotic surgery on decision making: perspectives of surgical teams. AMIA Annu Symp Proc.

[47] Sadri H, Fung-Kee-Fung M, Shayegan B, Garneau PY, Pezeshki P (2023). A systematic review of full economic evaluations of robotic-assisted surgery in thoracic and abdominopelvic procedures. J Robot Surg.

[48] Chahal B, Aydin A, Amin MS, Khan A, Khan MS, Ahmed K, Dasgupta P (2023). The learning curves of major laparoscopic and robotic procedures in urology: a systematic review. Int J Surg.

[49] Li J, Mao Y, Zhang J (2022). Maintenance and quality control of medical equipment based on information fusion technology. Comput Intell Neurosci.

[50] Wu JY, Yilmaz N, Tumerdem U, Kazanzides P (2021). Robot force estimation with learned intraoperative correction. 2021 International Symposium on Medical Robotics (ISMR).

[51] Zahid A, Ayyan M, Farooq M (2023). Robotic surgery in comparison to the open and laparoscopic approaches in the field of urology: a systematic review. J Robot Surg.

